# Supplementary material for: A rapid, multiplex digital PCR assay to detect gene variants and fusions in non‐small cell lung cancer
Source: Mol Oncol. 2023 Oct 4;17(11):2221–34. doi: 10.1002/1878-0261.13523 (PMC10620117; doi:10.1002/1878-0261.13523)
Supplement: Supplementary file 1 — Fig. S1. Multiplex digital PCR assay layout by instrument color channel. Fig. S2. Example multiplex digital PCR scatterplot for negative controls. Fig. S3. (A) Visualization of ROX reference dye variability across digital PCR arrays. (B) Passive reference dye quality control threshold. Fig. S4. Synthetic titration data for RET, ALK, and ROS1 with amplitude modulation chemistry and commercially available kit chemistry. Table S1. Assay layout for the three well multiplex NSCLC dPCR assay inclusive for 12 SNV and indel variants, 14 fusion variants, and MET exon 14 skipping. Table S2. Clinical sample metadata. Table S3. Fusion gBlock Sequences for in vitro transcription of synthetic RNA fusion molecules. Table S4. (A) Assay primer sequences. (B) Assay probe sequences. Table S5. Oligonucleotide primer–probe composition for amplitude modulation assay. Table S6. Design of contrived DNA and RNA sample experiments. Table S7. Optimized ROC thresholds of the target to the in‐well positive control. Table S8. Clinical sample molecular data. Table S9. The RNA fusion amplitude modulation assay benchmarks against a fusion RNA reference mix. Table S10. (A) Clinical sample results comparing dPCR to NGS. (B) Digital PCR can provide results where sequencing has gaps. [file MOL2-17-2221-s001.zip › Supplementary Figures and Tables.pdf]

Supporting Information

A rapid, multiplex digital PCR assay to detect gene variants and fusions in non-small cell lung cancer

Bryan Leatham[1], Katie McNall[1], Hari K.K. Subramanian[1], Lucien Jacky[1], John Alvarado[1], Dominic Yurk[1,3], Mimi Wang[1, 4], Donald C. Green[2], Gregory J. Tsongalis[2], Aditya Rajagopal[1,3,5], Jerrod J. Schwartz[1]

Addresses:
1 ChromaCode Inc, 2330 Faraday Ave, Suite 100, Carlsbad, CA 92008
2 Department of Pathology and Laboratory Medicine, Dartmouth Hitchcock Medical Center, 1 Medical Center Drive, Lebanon, New Hampshire 03756
3 California Institute of Technology, Department of Electrical Engineering, 1200 E California Blvd, Pasadena, CA 91125
4 Current address: Slack Technologies, 500 Howard St, San Francisco, CA 94105
5 Department of Biomedical Engineering, University of Southern California, Los Angeles, California 90089, United States

Corresponding author:
Jerrod J. Schwartz, 213-510-6252, jschwartz@chromacode.com

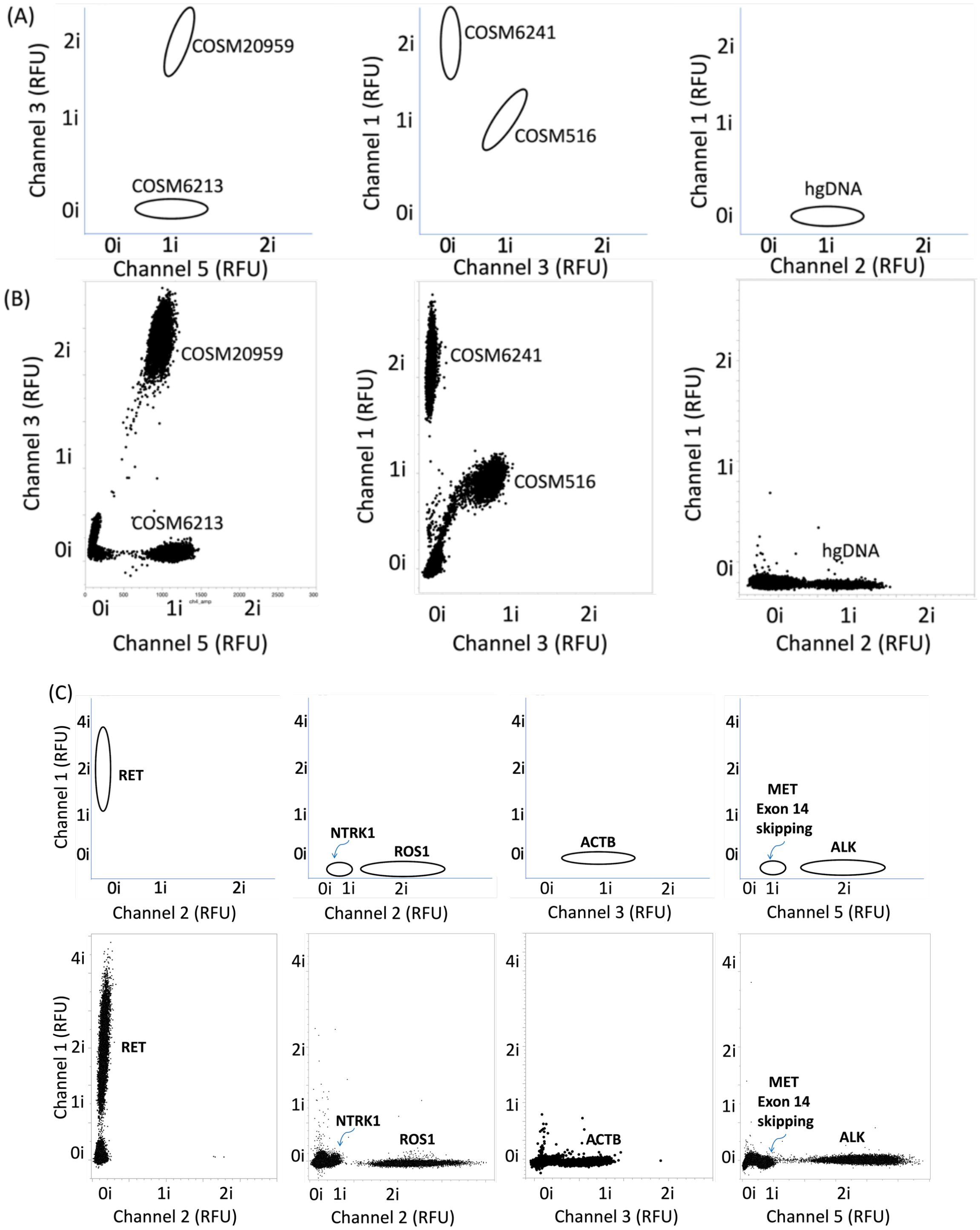

Figure S1. Multiplex digital PCR assay layout by instrument color channel. A) Illustrative locations on two-channel plot space where each variant-positive partitions are assigned for well #2 targets. B) Superimposed fluorescence scatterplots for synthetic targets profiled individually for *EGFR* S768I (COSM6241), *ERBB2* (COSM20959), *EGFR* L861Q (COSM6213), and *KRAS* G12C (COSM516). C) Approximate gate locations for RNA well #3 and example scatterplot showing the targets landing in the associated call window. RFU = Relative Fluorescence Units.

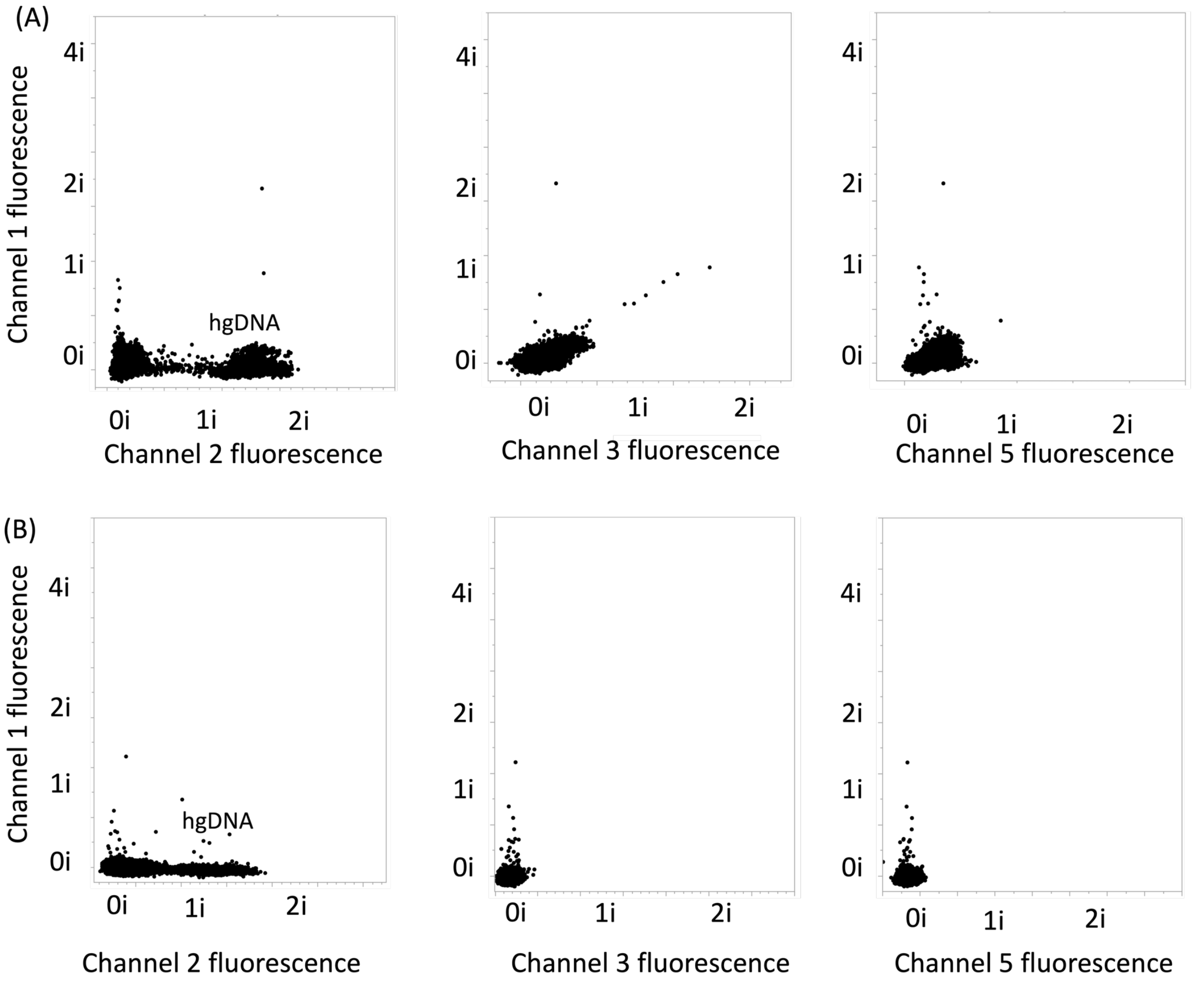

Figure S2. Example multiplex digital PCR scatterplot for negative controls. (A) Negative control (only human genomic DNA present) data for the plots shown in Figure 4. (B) The negative control for well #2.

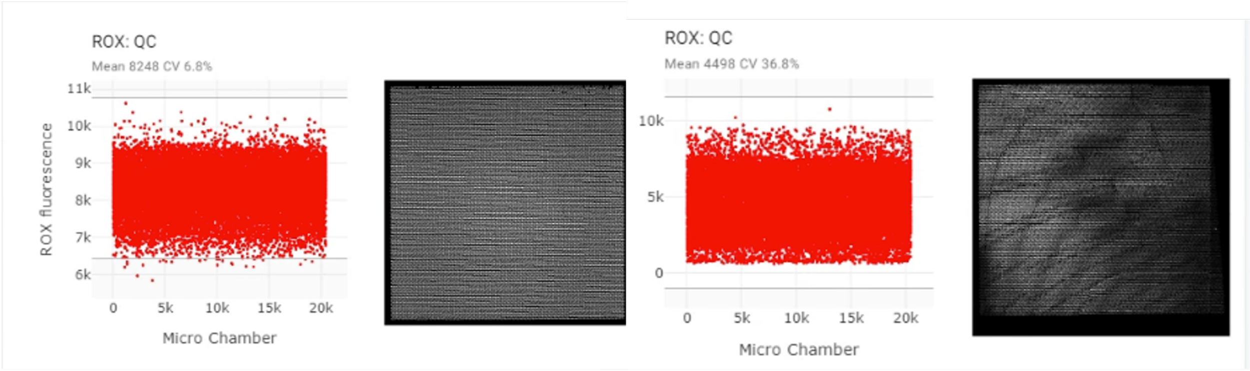

Example of a good QC array image and fluorescence distribution

Example of a failed well with streaks and dark patches in the QC array image

Figure S3A: Visualization of ROX reference dye variability across digital PCR arrays. Comparison of the QC data and raw image of a valid Absolute Q well (left) and a failed well (right).

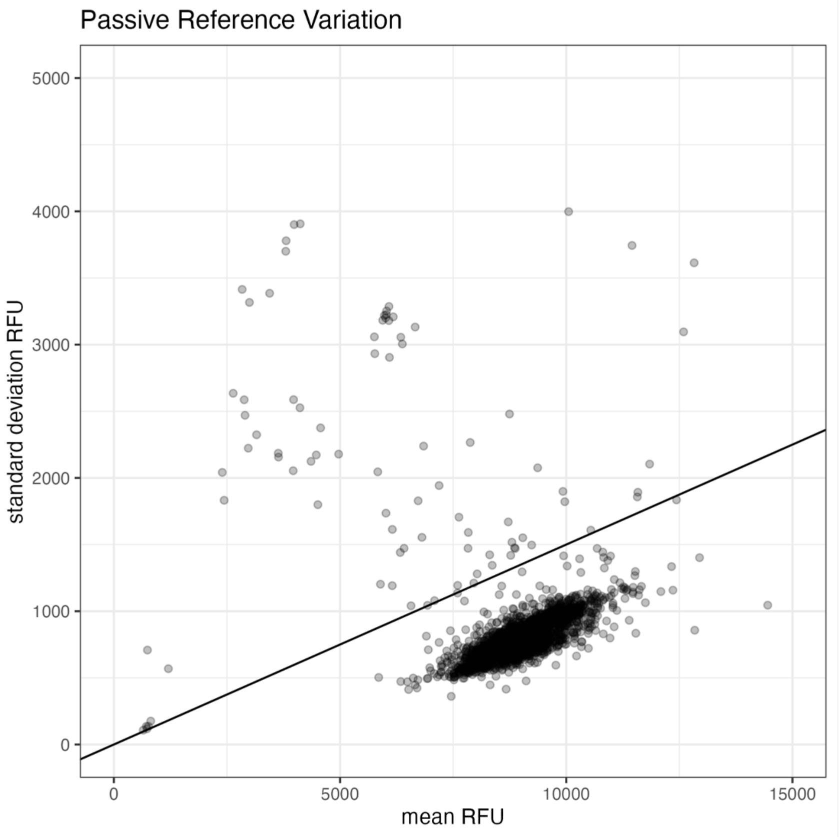

Figure S3B: Passive reference dye quality control threshold. Variation of the passive reference signal for every well in both the clinical and contrived data sets. The line represents the 15% coefficient of variation threshold; all wells above this threshold were excluded from the analysis.

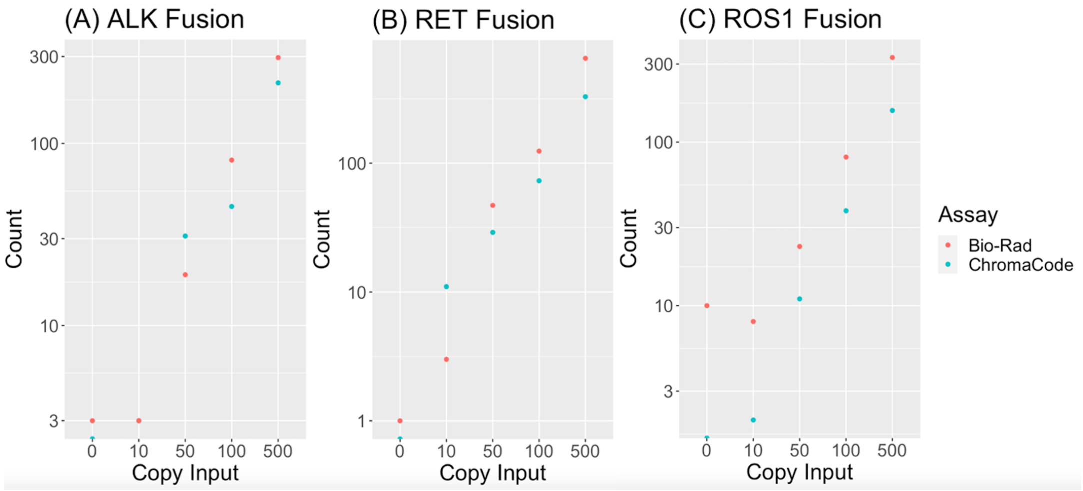

Figure S4: Synthetic titration data for *RET*, *ALK*, *ROS1* with amplitude modulation chemistry and commercially available kit chemistry.

Table S1. Assay layout for the three well NSCLC dPCR assay inclusive for 12 SNV and indel variants, 14 fusion variants, and *MET* exon 14 skipping.

Variant	Variant Type	Well	Legacy Mutation Identifier (COSM)	Genomic Mutation Identifier (COSV)	COSMIC Prevalence
EGFR L858R	SNV	1	COSM6224	COSV51765161	100%
EGFR T790M	SNV	1	COSM6240	COSV51765492	100%
KRAS G12C	SNV	2	COSM516	COSV55497469	100%
EGFR G719X	SNV	1	COSM6239<br>COSM6252<br>COSM6253	COSV51769339<br>COSV51767289<br>COSV51766606	100%
BRAF V600E	SNV	1	COSM476	COSV56056643	100%
EGFR S768I	SNV	2	COSM6241	COSV51768106	100%
EGFR L861Q	SNV	2	COSM6213	COSV51766344	100%
ERBB2 Y772_A775dup	Insertion	2	COSM20959	COSV54062409	54.7%
EGFR Exon 20 H773dup	Insertion	1	COSM12377	COSV51781591	8.1%
EGFR E746_A750del	Deletion	1	COSM6223	COSV51765119	50.8%
MET Exon 14	Exon Skipping	3	n/a	n/a	n/a
EML4-ALK	Fusion	3	COSF408<br>COSF409<br>COSF411<br>COSF474	n/a	87.6%
KIF5B-RET	Fusion	3	COSF1232<br>COSF1230<br>COSF1253<br>COSF1234	n/a	69.3%
CD74-ROS1<br>*SDC4-ROS1*<br>*EZR-ROS1*	Fusion	3	COSF1200<br>COSF1202<br>COSF1265<br>COSF1267	n/a	61.1%
TPM3-NTRK1<br>*MPRIP-NTRK1*	Fusion	3	COSF1329<br>n/a	n/a	17.3%

Table S2: Clinical sample metadata.
Table_S2_Clinical_Sample_Metadata.xlsx

Table S3: Fusion gBlock Sequences for in vitro transcription of synthetic RNA fusion molecules.

> MPRIP-NTRK1
TAATACGACTCACTATAGGGAGAATTAGCTCCCTCAAGGATGAGCTGCAGACGGCACTGCGGGACAAGAAGTACGCAAGT
GACAAGTACAAAGACATCTACACAGAGCTCAGCATCGCGAAGGCTAAGGCTGACTGTGACATCAGCAGGTTGAAGGAGCA
GCTCAAGGCTGCAACGGAAGCACTGGGGGAGAAGTCCCCTGACAGTGCCACGGTGTCCGGATATGGCCCGGCTGTGCT
GGCTCCAGAGGATGGGCTGGCCATGTCCCTGCATTTCATGACATTGGGTGGCAGCTCCCTGTCCCCCACCGAGGGCAAA
GGCTCTGGGCTCCAAGGCCACATCATCGAGAACCCACAATACTTCAGTGATGCCTGTGTTCACCACATCAAGCGCCGGGA
CATCGTGCTCAAGTGGGAGCTGGGGGAGGGCGCCTTTGGGAA

> EML4-ALK VAR 2
TAATACGACTCACTATAGGGAGAGTTTGTTCTGGATGCAGAAACCAGAGATCTAGTTTCTATCCACACAGACGGGAATGAA
CAGCTCTCTGTGATGCGCTACTCAATAGATGGTACCTTCCTGGCTGTAGGATCTCATGACAACTTTATTTACCTCTATGTAG
TCTCTGAAAATGGAAGAAAATATAGCAGATATGGAAGGTGCACTGGACATTCCAGCTACATCACACACCTTGACTGGTCCC
CAGACAACAAGTATATAATGTCTAACTCGGGAGACTATGAAATATTGTACTTGTACCGCCGGAAGCACCAGGAGCTGCAAG
CCATGCAGATGGAGCTGCAGAGCCCTGAGTACAAGCTGAGCAAGCTCCGCACCTCGACCATCATGACCGACTACAACCC
CAACTACTGCTTTGCTGGCAAGACCTCCTCCATCAGTGACCTGAAGGAGGTGCCGCGGAAAAACATCACCCTCATTCGGG
GTCTGGGCCATGGAGCCTTTGGGGAGGTG

> EML4-ALK VAR 3, iso a
TAATACGACTCACTATAGGGAGACGAGCATCACCTTCTCCCCAGCCCTCTTCACAACCTCTCCAAATACACAGACAAACTC
CAGAAAGCAAGAATGCTACTCCCACCAAAAGCATAAAACGACCATCACCAGCTGAAAAGTCACATAATTCTTGGGAAAATT
CAGATGATAGCCGTAATAAATTGTCGAAAATACCTTCAACACCCAAATTAATACCAAAAGTTACCAAAACTGCAGACAAGCA
TAAAGATGTCATCATCAACCAAGTGTACCGCCGGAAGCACCAGGAGCTGCAAGCCATGCAGATGGAGCTGCAGAGCCCT
GAGTACAAGCTGAGCAAGCTCCGCACCTCGACCATCATGACCGACTACAACCCCAACTACTGCTTTGCTGGCAAGACCTC
CTCCATCAGTGACCTGAAGGAGGTGCCGCGGAAAAACATCACCCTCATTCGGGGTCTGGGCCATGGAGCCTTTGGGGAG
GTGTATGAAGGCCAGGTGTCCGGAATGCCCA

> EML4-ALK VAR 3, iso b
TAATACGACTCACTATAGGGAGACGAGCATCACCTTCTCCCCAGCCCTCTTCACAACCTCTCCAAATACACAGACAAACTC
CAGAAAGCAAGAATGCTACTCCCACCAAAAGCATAAAACGACCATCACCAGCTGAAAAGTCACATAATTCTTGGGAAAATT
CAGATGATAGCCGTAATAAATTGTCGAAAATACCTTCAACACCCAAATTAATACCAAAAGTTACCAAAACTGCAGACAAGCA
TAAAGATGTCATCATCAACCAAGCAAAAATGTCAACTCGCGAAAAAAACAGCCAAGTGTACCGCCGGAAGCACCAGGAGC
TGCAAGCCATGCAGATGGAGCTGCAGAGCCCTGAGTACAAGCTGAGCAAGCTCCGCACCTCGACCATCATGACCGACTA
CAACCCCAACTACTGCTTTGCTGGCAAGACCTCCTCCATCAGTGACCTGAAGGAGGTGCCGCGGAAAAACATCACCCTCA
TTCGGGGTCTGGGCCATGGAGCCTTTGGGG

> KIF5B-RET; K16:R12
TAATACGACTCACTATAGGGAGAGCATCTTTACTAAAAGACCTTGCAGAAATAGGAATTGCTGTGGGAAATAATGATGTAAA
GCAGCCTGAGGGAACTGGCATGATAGATGAAGAGTTCACTGTTGCAAGACTCTACATTAGCAAAATGAAGTCAGAAGTAAA
ACCATGGTGAAACGTTGCAAGCAGTTAGAAAGCACACAAACTGAGAGCAACAAAAAAATGGAAGAAAATGAAAAGGAGTTA
GCAGCATGTCAGCTTCGTATCTCTCAAGAGGATCCAAAGTGGGAATTCCCTCGGAAGAACTTGGTTCTTGGAAAAACTCTA
GGAGAAGGCGAATTTGGAAAAGTGGTCAAGGCAACGGCCTTCCATCTGAAAGGCAGAGCAGGGTACACCACGGTGGCCG
TGAAGATGCTGAAAGAGAACGCCTCCCCGAGTGAGCTGCGAGACCTGCTGTCAGAGTTCAACGTCCTGAAGCA

> KIF5B-RET; K22:R12
TAATACGACTCACTATAGGGAGAAAGCAAAAGCAAAACTTATTACTGATCTTCAAGACCAAAACCAGAAAATGATGTTAGAG
CAGGAACGTCTAAGAGTAGAACATGAGAAGTTGAAAGCCACAGATCAGGAAAAGAGCAGAAAACTACATGAACTTACGGTT
ATGCAAGATAGACGAGAACAAGCAAGACAAGACTTGAAGGGTTTGGAAGAGACAGTGGCAAAAGAACTTCAGACTTTACA
CAACCTGCGCAAACTCTTTGTTCAGGACCTGGCTACAAGAGTTAAAAAGGAGGATCCAAAGTGGGAATTCCCTCGGAAGA
ACTTGGTTCTTGGAAAAACTCTAGGAGAAGGCGAATTTGGAAAAGTGGTCAAGGCAACGGCCTTCCATCTGAAAGGCAGA
GCAGGGTACACCACGGTGGCCGTGAAGATGCTGAAAGAGAACGCCTCCCCGAGTGAGCTGCGAGACCTGCTGT

>KIF5B-RET; K23:R12
TAATACGACTCACTATAGGGAGAAAAGAGCAGAAAACTACATGAACTTACGGTTATGCAAGATAGACGAGAACAAGCAAGA
CAAGACTTGAAGGGTTTGGAAGAGACAGTGGCAAAAGAACTTCAGACTTTACACAACCTGCGCAAACTCTTTGTTCAGGAC
CTGGCTACAAGAGTTAAAAAGAGTGCTGAGATTGATTCTGATGACACCGGAGGCAGCGCTGCTCAGAAGCAAAAAATCTC
CTTTCTTGAAAATAATCTTGAACAGCTCACTAAAGTGCACAAACAGGAGGATCCAAAGTGGGAATTCCCTCGGAAGAACTT
GGTTCTTGGAAAAACTCTAGGAGAAGGCGAATTTGGAAAAGTGGTCAAGGCAACGGCCTTCCATCTGAAAGGCAGAGCAG
GGTACACCACGGTGGCCGTGAAGATGCTGAAAGAGAACGCCTCCCCGAGTGAGCTGCGAGACCTGCTGTCAGAG

>CD74, exon 6:ROS1, exon 34
TAATACGACTCACTATAGGGAGACCCATGCAGAATGCCACCAAGTATGGCAACATGACAGAGGACCATGTGATGCACCTG
CTCCAGAATGCTGACCCCCTGAAGGTGTACCCGCCACTGAAGGGGAGCTTCCCGGAGAACCTGAGACACCTTAAGAACA
CCATGGAGACCATAGACTGGAAGGTCTTTGAGAGCTGGATGCACCATTGGCTCCTGTTTGAAATGAGCAGGCACTCCTTG
GAGCAAAAGCCCACTGACGCTCCACCGAAAGATGATTTTTGGATACCAGAAACAAGTTTCATACTTACTATTATAGTTGGAA

TATTTCTGGTTGTTACAATCCCACTGACCTTTGTCTGGCATAGAAGATTAAAGAATCAAAAAAGTGCCAAGGAAGGGGTGA
CAGTGCTTATAAACGAAGACAAAGAGTTGGCTGAGCTGCGAGGTCTGGCAGCCGGAGTAGGCCTGGCTAATGCCT

>CD74, exon 6:ROS1, exon 32
TAATACGACTCACTATAGGGAGACCCCTGAAGGTGTACCCGCCACTGAAGGGGAGCTTCCCGGAGAACCTGAGACACCTT
AAGAACACCATGGAGACCATAGACTGGAAGGTCTTTGAGAGCTGGATGCACCATTGGCTCCTGTTTGAAATGAGCAGGCA
CTCCTTGGAGCAAAAGCCCACTGACGCTCCACCGAAAGCTGGAGTCCCAAATAAACCAGGCATTCCCAAATTACTAGAAG
GGAGTAAAAATTCAATACAGTGGGAGAAAGCTGAAGATAATGGATGTAGAATTACATACTATATCCTTGAGATAAGAAAGAG
CACTTCAAATAATTTACAGAACCAGAATTTAAGGTGGAAGATGACATTTAATGGATCCTGCAGTAGTGTTTGCACATGGAAG
TCCAAAAACCTGAAAGGAATATTTCAGTTCAGAGTAGTAGCTGCAAATAATCTAGGGTTTGGTGAATATAGTGGAATC

> SDC4, exon 2:ROS1, exon 32
TAATACGACTCACTATAGGGAGACGTAGGCGGAGTCGCCGAGTCGATCCGAGAGACTGAGGTCATCGACCCCCAGGACC
TCCTAGAAGGCCGATACTTCTCCGGAGCCCTACCAGACGATGAGGATGTAGTGGGGCCCGGGCAGGAATCTGATGACTT
TGAGCTGTCTGGCTCTGGAGATCTGGCTGGAGTCCCAAATAAACCAGGCATTCCCAAATTACTAGAAGGGAGTAAAAATTC
AATACAGTGGGAGAAAGCTGAAGATAATGGATGTAGAATTACATACTATATCCTTGAGATAAGAAAGAGCACTTCAAATAAT
TTACAGAACCAGAATTTAAGGTGGAAGATGACATTTAATGGATCCTGCAGTAGTGTTTGCACATGGAAGTCCAAAAACCTG
AAAGGAATATTTCAGTTCAGAGTAGTAGCTGCAAATAATCTAGGGTTTGGTGAATATAGTGGAATCAGTGAGAATATTA

> TPM3-NTRK1
TAATACGACTCACTATAGGGAGACCTGAAGTGTCTGAGTGCTGCTGAAGAAAAGTACTCTCAAAAAGAAGATAAATATGAG
GAAGAAATCAAGATTCTTACTGATAAACTCAAGGAGGCAGAGACCCGTGCTGAGTTTGCTGAGAGATCGGTAGCCAAGCT
GGAAAAGACAATTGATGACCTGGAAGACACTAACAGCACATCTGGAGACCCGGTGGAGAAGAAGGACGAAACACCTTTTG
GGGTCTCGGTGGCTGTGGGCCTGGCCGTCTTTGCCTGCCTCTTCCTTTCTACGCTGCTCCTTGTGCTCAACAAATGTGGA
CGGAGAAACAAGTTTGGGATCAACCGCCCGGCTGTGCTGGCTCCAGAGGATG

>KIF5B-RET; K15:R12
TAATACGACTCACTATAGGGAGACAGGCCCTAGAAGAACTTGCTGTCAATTATGATCAGAAGTCTCAGGAAGTTGAAGACA
AAACTAAGGAATATGAATTGCTTAGTGATGAATTGAATCAGAAATCGGCAACTTTAGCGAGTATAGATGCTGAGCTTCAGAA
ACTTAAGGAAATGACCAACCACCAGAAAAAACGAGCAGCTGAGATGATGGCATCTTTACTAAAAGACCTTGCAGAAATAGG
AATTGCTGTGGGAAATAATGATGTAAAGGAGGATCCAAAGTGGGAATTCCCTCGGAAGAACTTGGTTCTTGGAAAAACTCT
AGGAGAAGGCGAATTTGGAAAAGTGGTCAAGGCAACGGCCTTCCATCTGAAAGGCAGAGCAGGGTACACCACGGTGGCC
GTGAAGATGCTGAAAGAGAACGCCTCCCCGAGTGAGCTGCGAGACCTGCTGTCAGAGTTCAACGTCCTGAAGCAG

> EZR, exon 10:ROS1, exon 34
TAATACGACTCACTATAGGGAGACGCCGCAGGAAGCCTGACACCATCGAGGTGCAGCAGATGAAGGCCCAGGCCCGGGA
GGAGAAGCATCAGAAGCAGCTGGAGCGGCAACAGCTGGAAACAGAGAAGAAAAGGAGAGAAACCGTGGAGAGAGAGAA
AGAGCAGATGATGCGCGAGAAGGAGGAGTTGATGCTGCGGCTGCAGGACTATGAGGAGAAGACAAAGAAGGCAGAGAG
AGATGATTTTTGGATACCAGAAACAAGTTTCATACTTACTATTATAGTTGGAATATTTCTGGTTGTTACAATCCCACTGACCT
TTGTCTGGCATAGAAGATTAAAGAATCAAAAAAGTGCCAAGGAAGGGGTGACAGTGCTTATAAACGAAGACAAAGAGTTGG
CTGAGCTGCGAGGTCTGGCAGCCGGAGTAGGCCTGGCTAATGCCTGCTATGCAATACATACTCTTCCAACCCAAGAGGAG
ATTGAAAATCTTCCTGCCTTCCCTCGGGAAAAAC

> EML4-ALK variant 1, E13:A20
TAATACGACTCACTATAGGGAGACCATAATTACATGCGGTAAATCTCATATTTTCTTCTGGACCTGGAGCGGCAATTCACTA
ACAAGAAAACAGGGAATTTTTGGGAAATATGAAAAGCCAAAATTTGTGCAGTGTTTAGCATTCTTGGGGAATGGAGATGTT
CTTACTGGAGACTCAGGTGGAGTCATGCTTATATGGAGCAAAACTACTGTAGAGCCCACACCTGGGAAAGGACCTAAAGT
GTACCGCCGGAAGCACCAGGAGCTGCAAGCCATGCAGATGGAGCTGCAGAGCCCTGAGTACAAGCTGAGCAAGCTCCG
CACCTCGACCATCATGACCGACTACAACCCCAACTACTGCTTTGCTGGCAAGACCTCCTCCATCAGTGACCTGAAGGAGG
TGCCGCGGAAAAACATCACCCTCATTCGGGGTCTGGGCCATGGAGCCTTTGGGGAGGTGTATGAAGGCCAGGTGTCCGG
AATGCCCAACGACCCAAGCCCCCTGCAAGTGGC

Table S4A. Assay primer sequences.

Vendor	Name	Type	Hg38 coordinate start	Sequence 5'-3'
**IDT**	L858R_F	Forward	Chr7: 55191744	GCTTGGTGCACCGCGACCTG
**IDT**	L858R_R	Reverse	Chr7:55191821	CGCACCCTGCAGTTTGGCAC
**IDT**	E746_A750del_F	Forward	Chr7:55174753	AATTCCCGTCGCTATCAAAAC
**IDT**	E746_A750del_R	Reverse	Chr7:55174830	ACCCCCACACAGCAAAGC
**IDT**	T790M_F	Forward	Chr7:55181433	GCAGGTACTGGGAGCCAAT
**IDT**	T790M_Insertion_R	Reverse	Chr7:55181297	CTACGTGATGGCCAGCG
**IDT**	V600E_F	Forward	Chr7: 140753316	CCCACTCCATCGAGATTTGT
**IDT**	V600E_R	Reverse	Chr7: 140753379	ACTACACCTCAGATATATTTCTTCATG
**IDT**	G719S_F	Forward	Chr7: 55173990	GAATTCAAAAAGATCAAAGTGCTAA
**IDT**	G719C_F	Forward	Chr7: 55173990	GAATTCAAAAAGATCAAAGTGCTAT
**IDT**	G719A_F	Forward	Chr7:55173993	TTCAAAAAGATCAAAGTGCTGAC
**IDT**	G719X_R	Reverse	Chr7: 55174112	AAATATACAGCTTGCAAGGAC
**IDT**	G719X_B	Blocker	Chr7:55173993	TTCAAAAAGATCAAAGTGCTGAG/3Phos/
**IDT**	EGFR-Exon2-F	Forward	Chr7:55142288	TGCCAAGGCACGAGTAACAAG
**IDT**	EGFR-Exon2-R	Reverse	Chr7:55142375	TCCAAATTCCCAAGGACCAC
**IDT**	G12C_F	Forward	Chr12: 25245309	TCTGAATTAGCTGTATCGTCAAGG
**IDT**	G12C_R	Reverse	Chr12: 25245379	ATTATAAGGCCTGCTGAAAATGACT
**IDT**	G12C_B	Blocker	Chr12:25245345	TGGAGCTGGTGGCGT/3Phos/
**IDT**	HER2- Y772_A775dup_R	Reverse	Chr17: 39724824	ATGGTCTAAGAGGCAGCCATAG
**IDT**	HER2-Y772_A775dup_F	Forward	Chr17: 39724724	AGGAAGCATACGTGATGGCATA
**IDT**	S768I_R2	Reverse	Chr7:55181312	CACGTGGGGGTTGTCCACCA
**IDT**	S768I_F2	Forward	Chr7: 55181218	CCTGGAAGGGGTCCATGTGC
**IDT**	L861Q_F	Forward	Chr7:55191816	GATTTTGGGCTGGCCAAATA
**IDT**	L861Q_R	Reverse	Chr7: 55191935	TGTGTTAAACAATACAGCTAGTGG
**IDT**	EML4-ALK variant 1	Forward	Chr2: 42295496	CACCTGGGAAAGGACCTAAAG
**IDT**	EML4-ALK variant 1	Reverse	Chr2: 29223453	AGCTTGCTCAGCTTGTACTC
**IDT**	EML4-ALK variant 2	Forward	Chr2: 42325529	CTCGGGAGACTATGAAATATTGTACT
**IDT**	EML4-ALK variant 2	Reverse	Chr2:29223474	GGCTCTGCAGCTCCATC
**IDT**	EML4-ALK variant 3/4 iso	Forward	Chr2: 42264706	GACAAGCATAAAGATGTCATCATCAAC
**IDT**	EML4-ALK variant 4	Forward	Chr2: 42264712	AGATGTCATCATCAACCAAGCA
**IDT**	KIF5B-RET; K15:R12	Forward	Chr10: 32028553	TCGGCAACTTTAGCGAGTATAG
**IDT**	KIF5B-RET; K15:R12	Reverse	Chr10: 43116601	CCAAGTTCTTCCGAGGGAAT
**IDT**	KIF5B-RET; K16:R12	Forward	Chr10: 32022902	CACACAAACTGAGAGCAACAAA
**IDT**	KIF5B-RET; K22:R12	Forward	Chr10: 32018504	TGAAGGGTTTGGAAGAGACAG
**IDT**	MPRIP-NTRK1; M21:N14	Forward	Chr17:17177348	CTCAAGGCTGCAACGGAAG
**IDT**	MPRIP-NTRK1; M21:N14	Reverse	Chr1:156875540	CATGGCCAGCCCATCCT
**IDT**	TPM3-NTRK1	Forward	Chr1:154170420	CGGTAGCCAAGCTGGAAA
**IDT**	TPM3-NTRK1	Reverse	Chr1:156874931	AAGGAAGAGGCAGGCAAAG
**IDT**	CD74-ROS1; C6:R34	Reverse	Chr6: 117324332	GACAAAGGTCAGTGGGATTGTA
**IDT**	CD74-ROS1; C6:R34	Forward	Chr5: 150404750	AAGGTCTTTGAGAGCTGGATG

**IDT**	CD74-ROS1; C6:R32	Reverse	Chr6: 117329416	TTGGGAATGCCTGGTTTATTTG
**IDT**	SDC4-ROS1; S2:R32	Forward	Chr20: 45335865	TCCTAGAAGGCCGATACTTCTC
**IDT**	SDC4-ROS1; S2:R32	Reverse	Chr6: 117329425	CCTGGTTTATTTGGGACTCCAG
**IDT**	EZR-ROS1; E10:R34	Forward	Chr6: 158770842	GAAACCGTGGAGAGAGAGAAAG
**IDT**	EZR-ROS1; E10:R34	Reverse	Chr6:117324331	AGACAAAGGTCAGTGGGATTG
**IDT**	MET Exon14 WT	Forward	Chr7: 116771920	CGAAGTGTAAGCCCAACTACA
**IDT**	MET Exon15 WT	Reverse	Chr7: 116771982	GAATTAGGAAACTGATCTTCTGG
**IDT**	Exon14skip F	Forward	Chr7:116771612	ATGGGTTTTTCCTGTGGCTGAA
**IDT**	Exon14skip R	Reverse	Chr7: 116774894	GCATGAACCGTTCTGAGATGAA
**IDT**	ACTB F control	Forward	Chr7:5528175	CCTTCCTTCCTGGGCATGGAGTC
**IDT**	ACTB R control	Reverse	Chr7: 5528088	AGACAGCACTGTGTTGGCGT

Table S4B. Assay probe sequences.

Vendor	Name	Probe Channel	Hg38 coordinate start	Sequence 5'-3'
**IDT**	L858R_P	1	Chr7: 55191766	AGCCAGGAACGTACTGGTGAAAACACCGCA
**TFS**	L858R_P	3	Chr7: 55191766	AGCCAGGAACGTACTGGTGAAAACACCGCA
**IDT**	E746_A750del_P	1	Chr7: 55174801	CCAACAAGGAAATCCTCGATGTGAGTTTCTG
**IDT**	T790M_P	1	Chr7:55181372	TGAGCTGCATGATG
**IDT**	T790M_P	5	Chr7:55181372	TGAGCTGCATGATG
**IDT**	EGFR-E20-Insertion-CAC	2	Chr7:55181321	CC+C +C+A+C CAC G (+ is LNA)
**TFS**	V600E_P	3	Chr7: 140753353	ATCACCTATTTTTACTGTGAGGTCTT
**IDT**	G719X_P_1	5	Chr7: 55174039	ATAAGGTAAGGTCCCTGGCACA
**IDT**	EGFR-Exon2-P	2	Chr7: 55142341	TCTCAGCCTCCAGAGGATGTTCAATAACT
**IDT**	KRASG12C	1	Chr12:25245343	TGGAGCTTGTGGCGT
**TFS**	KRASG12C	3	Chr12:25245343	TGGAGCTTGTGGCGT
**TFS**	HER2- Y772_A775dup probe	3	Chr17: 39724772	TTCTGGGCATCTGCCTGACATCC
**IDT**	HER2- Y772_A775dup probe	5	Chr17: 39724772	TTCTGGGCATCTGCCTGACATCC
**IDT**	S768I_P2	1	Chr7: 55181274	TGCCTCTCCCTCCCTCCAGGAAGCC
**IDT**	L861Q_P	5	Chr7: 55191855	ACCATGCAGAAGGAGGCAAAGTAAGG
**IDT**	KIF5B-RET; K15:R12	1	Chr10: 32028484	AACGAGCAGCTGAGATGATGGCAT
**IDT**	KIF5B-RET; K16:R12	1	Chr10: 32022858	AGGAGTTAGCAGCATGTCAGCTTCG
**IDT**	KIF5B-RET; K22:R12	1	Chr10: 32018335	AGGTCCTGAACAAAGAGTTTGCGC
**IDT**	CD74-ROS1; C6:R34	2	Chr5: 150404709	TGAAATGAGCAGGCACTCCTTGGA
**IDT**	SDC4-ROS1; S2:R32	2	Chr20: 45335838	AGCCCTACCAGACGATGAGGATGT
**IDT**	EZR-ROS1; E10:R34	2	Chr6: 158770810	ATGCGCGAGAAGGAGGAGTTGATG
**IDT**	MPRIP-NTRK1; M21:N14	2	Chr17: 17177391	AGTGCCACGGTGTCCGGATATG
**IDT**	TPM3-NTRK1	2	Chr1: 156874593	CCCGGTGGAGAAGAAGGACGAAAC
**IDT**	EML4-ALK variant 1	5	Chr2: 29223482	CAGCTCCATCTGCATGGCTTGC
**IDT**	EML4-ALK variant 2	5	Chr2: 29223494	ACCAGGAGCTGCAAGCCATG
**IDT**	Exon14 skip probe (spans 13-15)	5	Chr8: 119489556	GGAAACTGATCTTTAATTTGCTTTCTC
**IDT**	Exon14-15 WT probe	3	Chr7: 116771943	AATGGTTTCAAATGAATCTGTAGAC
**IDT**	ACTB probe	3	Chr7: 5528112	TGACGTGGACATCCGCAAAGAC

Table S5. Oligonucleotide primer-probe composition for amplitude modulation assay. Component name suffixes identify the type of oligo used ('_F' = forward primer; '_R' = reverse primer; '_B' = blocker and '_P' = probe).

DNA Well #1

Component	Final concentration in dPCR reaction (nM)
L858R_F	200
L858R_R	200
E746_A750del_F	200
E746_A750del_R	200
T790M_F	200
T790M_Insertion_R	200
V600E_F	200
V600E_R	200
G719S_F	200
G719C_F	200
G719A_F	200
G719X_R	200
G719X_B	200
EGFR-Exon2-F	200
EGFR-Exon2-R	200
L858R_P	22.4
L858R_P	20
E746_A750del_P	20
T790M_P	18.3
T790M_P	20
EGFR-E20-Insertion-CAC_P	20
V600E_P	5.8
G719X_P_1	6.7
EGFR-Exon2-P	8.5

DNA Well #2

Component	Final concentration in dPCR reaction (nM)
EGFR-Exon2-F	300
EGFR-Exon2-R	300
G12C_F	300
G12C_R	300
G12C_B	300
HER2- Y772_A775dup_R	300
HER2-Y772_A775dup_F	300
S768I_R2	300
S768I_F2	300
L861Q_F	300
L861Q_R	300
EGFR-Exon2-P	20
KRASG12C_P	18
KRASG12C_P	18.6
HER2- Y772_A775dup probe	20
HER2- Y772_A775dup probe	20
S768I_P2	20
L861Q_P	12

RNA Well #3

Component	Final Concentration in dPCR Reaction (nM)
EML4-ALK variant 1 F	300
EML4-ALK variant 1 R	900
EML4-ALK variant 2 F	300
EML4-ALK variant 2 R	300
EML4-ALK variant 3/4 iso F2	300
EML4-ALK variant 4 F	600
KIF5B-RET; K15:R12 (F2)	300
KIF5B-RET; K15:R12 (R1)	1200
KIF5B-RET; K16:R12 (F2)	300

KIF5B-RET; K22:R12 (F2)	600
MPRIP (ex 21) - NTRK1 (ex 14) (F2)	300
MPRIP (ex 21) - NTRK1 (ex 14) (R2)	300
TPM3-NTRK1 (F2)	300
TPM3-NTRK1 (R2)	300
CD74, ex6:ROS1, ex34 (R2)	300
CD74, ex6:ROS1, ex34 (F1)	600
CD74, ex6:ROS1, ex32 (R1)	300
SDC4, ex2:ROS1, ex32 (F3)	300
SDC4, ex2:ROS1, ex32 (R3)	300
EZR, ex10:ROS1, ex34 (F1)	300
EZR, ex10:ROS1, ex34 (R1)	300
Exon14 WT F	300
Exon15 WT R	300
Exon14skip F (exon13)	300
Exon14skip R (exon15)	300
ACTB F control	300
ACTB R control	300
KIF5B-RET; K15:R12 (P2)	144
KIF5B-RET; K16:R12 (P2)	107
KIF5B-RET; K22:R12 (P1)	75
KIF5B-RET; K22:R12 (P1)	125
CD74, ex6:ROS1, ex34 (P1)	95
CD74, ex6:ROS1, ex34 (P1)	75
SDC4, ex2:ROS1, ex32 (P3)	116
EZR, ex10:ROS1, ex34 (P1)	101
MPRIP (ex 21) - NTRK1 (ex 14) (P1)	12
TPM3-NTRK1 (P1)	13
EML4-ALK variant 1 P	84
EML4-ALK variant 1 P	75
EML4-ALK variant 1 P	120
EML4-ALK variant 2 P	96
Exon14skip probe (spans 13-15)	29
Exon14-15 WT probe	392
ACTB probe	90

Table S6. Design of contrived DNA and RNA sample experiments. Each reportable in Table S1 was tested one at a time by combining wild type human biological genomic DNA and synthetic oligonucleotides containing each variant of interest. In addition, combinations of the most common variant combinations (*EGFR* L858R + *EGFR* T790M, *EGFR* T790M + *EGFR* Exon 19 del, *EGFR* Exon 19 del + *EGFR* L858R, and all three of these together) were tested under the same copy number conditions.

**DNA Background Type**	**Total mass of background DNA (from Absolute Q)**	Copy number of synthetic variant DNA (gBlock)	**Replicates at each copy number**
FFPE DNA	21 ng	2320	3
		1160	3
		464	3
		232	4
		116	4
		58	4

**RNA Background Type**	**ACTB copy number of background RNA (from Absolute Q)**	**Copy number of RNA Fusion (IVT product)**	**Replicates at each copy number**
FFPE RNA	5000	5000	3-10
		7500	3-10
		10000	3-10
		11250	10

Table S7. Optimized ROC thresholds of the target to the in-well positive control. Controls are *EGFR* Exon 2 and *ACTB* for the DNA and RNA assays, respectively.

Well	Target	Threshold
1	*EGFR* L858R	0.004
1	*EGFR* T790M	0.002
1	*EGFR* E746_A750del	0.0128
1	*BRAF* V600E	0.0126
1	*EGFR* G719S	0.012
2	*EGFR* H773dup	0.0047
2	*KRAS* G12C	0.0008
2	*ERBB2* Y772_A775dup	0.0746
2	*EGFR* S768I	0.0015
2	*EGFR* L861Q	0.0076
3	*EML4-ALK*	0.1096
3	*MET* Exon14	0.2474
3	*KIF5B-RET*	0.6988
3	*CD74-ROS1*	0.0036
3	*TMP3-NTRK1*	0.0026

Table S8: Clinical sample molecular data.

Table_S8_Clinical_Sample_Molecular_Data.xlsx

Table S9. The RNA fusion amplitude modulation assay benchmarks against a fusion RNA reference mix. The comparison was done using a commercially available fusion RNA reference mix (SeraSeq Fusion RNA Mix v4, 0710-0497, SeraCare) (number of replicates = 4). The reference and the assay shared inclusivity for *CD74-ROS1*, *EML4-ALK* var 1, and *MET* Exon 14 skipping; they did not share inclusivity for *RET*, *NTRK1*, *ACTB*, or *MET* wild type.

Channel	Variant	Amplitude modulation measured concentration (cp/μL)	Certificate of Analysis concentration (cp/μL)
Channel 2	*CD74-ROS1*; C6:R34	104.5	113.4
Channel 5	*MET* Ex 14 Skip	153.6	125.4
Channel 5	*EML4-ALK*, var 1	57.6	86.5

Table S10A. Clinical sample results comparing dPCR to NGS. All samples passed NGS QC and were positive in either NGS or dPCR.

Sample_ID	dPCR_Results	PCR_VAF	NGS_Results	NGS_VAF
6764-JS-0001	*EGFR* E746_A750del	45.8%	*EGFR* E746_A750del	46.0%
6764-JS-0002	*EGFR* E746_A750del	22.0%	*EGFR* E746_A750del	19.0%
6764-JS-0015	*EGFR* E746_A750del	73.4%	*EGFR* E746_A750del	49.0%
6764-JS-0055	*EGFR* H773dup	1.4%	Coverage gaps	NA
6764-JS-0072	*EGFR* L858R	15.5%	*EGFR* L858R	23.0%
DH-EGFR-10	*EGFR* E746_A750del	23.6%	*EGFR* E746_A750del	21.2%
DH-EGFR-12	*EGFR* E746_A750del	39.0%	*EGFR* E746_A750del	35.0%
DH-EGFR-16	*EGFR* E746_A750del	10.3%	*EGFR* E746_A750del	7.9%
DH-EGFR-18	*EGFR* L858R	2.3%	Not detected	NA
DH-EGFR-18	*EGFR* E746_A750del	5.96%	*EGFR* E746_A750del	35.6%
DH-EGFR-2	*EGFR* L858R	12.1%	*EGFR* L858R	11.0%
DH-EGFR-20	*EGFR* L858R Invalid ONC2	87.0%	*EGFR* L858R	73.8%
DH-EGFR-22	*EGFR* L858R	20.6%	*EGFR* L858R	11.5%
DH-EGFR-24	*EGFR* L858R	4.1%	*EGFR* L858R	2.9%
DH-EGFR-26	*EGFR* L858R	5.6%	*EGFR* L858R	3.9%
DH-EGFR-28	*EGFR* E746_A750del	73.3%	*EGFR* E746_A750del	35.3%
DH-EGFR-34	*EGFR* G719X	7.6%	Not detected	NA
DH-EGFR-36	*EGFR* E746_A750del	1.1%	Not detected	NA
DH-EGFR-40	*EGFR* L858R Invalid ONC2	31.5%	*EGFR* L858R	10.4%
DH-EGFR-44	*EGFR* E746_A750del Invalid ONC2	47.5%	*EGFR* E746_A750del	39.5%
DH-EGFR-46	*EGFR* L858R	10.9%	*EGFR* L858R	9.4%
DH-EGFR-48	*EGFR* T790M	6.8%	*EGFR* T790M	65.2%
DH-EGFR-48	*EGFR* G719X	2.0%	Not detected	NA
DH-EGFR-50	*EGFR* E746_A750del	134.0%	*EGFR* E746_A750del	11.2%
DH-EGFR-6	*EGFR* L858R	19.8%	*EGFR* L858R	8.3%
DH-EGFR-8	*EGFR* L858R	9.7%	*EGFR* L858R	6.5%

Table S10B. Digital PCR can provides results where sequencing has gaps. Clinical dPCR results for FFPE samples that either failed to sequence or had gaps in sequence coverage, while the dPCR assay generated valid results.

Sample_ID	Age	dPCR_Results	dPCR_VAF	DNA_NGS_Result
6764-JS-0004	< 3 yrs	*EGFR* E746_A750del	34.2%	Coverage gaps
6764-JS-0005	< 3 yrs	*EGFR* E746_A750del	76.1%	Coverage gaps
6764-JS-0010	< 3 yrs	None Detected		QNS
6764-JS-0011	< 3 yrs	*EGFR* E746_A750del	73.5%	Coverage gaps
6764-JS-0012	< 3 yrs	*EGFR* E746_A750del	23.7%	Coverage gaps
6764-JS-0013	< 3 yrs	None Detected		Coverage gaps
6764-JS-0014	< 3 yrs	*EGFR* T790M EGFR E746_A750del Invalid ONC2	0.4% 25.8%	Coverage gaps
6764-JS-0049	>=15 yrs	None Detected		QNS
6764-JS-0055	>=15 yrs	*EGFR* H773dup	1.4%	Coverage gaps
6764-JS-0056	>=15 yrs	None Detected		Coverage gaps
6764-JS-0057	>=15 yrs	*KRAS* G12C	9.7%	QNS
6764-JS-0062	>=15 yrs	None Detected		Coverage gaps
6764-JS-0065	>=15 yrs	None Detected		QNS
6764-JS-0068	>=15 yrs	None Detected		QNS
6764-JS-0069	>=15 yrs	None Detected		QNS
6764-JS-0074	>=15 yrs	None Detected		QNS
6764-JS-0078	>=15 yrs	None Detected		Coverage gaps
6764-JS-0079	>=15 yrs	None Detected		Coverage gaps
6764-JS-0084	>=15 yrs	None Detected		QNS
6764-JS-0085	>=15 yrs	*KRAS* G12C	1.2%	QNS
6764-JS-0086	>=15 yrs	*KRAS* G12C	1.1%	QNS
6764-JS-0090	>=15 yrs	*KRAS* G12C	5.9%	QNS
